# Supplementary material for: Propofol-based total intravenous anesthesia is associated with better survival than desflurane anesthesia in robot-assisted radical prostatectomy
Source: PLoS One. 2020 Mar 17;15(3):e0230290. doi: 10.1371/journal.pone.0230290 (PMC7077845; doi:10.1371/journal.pone.0230290)
Supplement: Attachment — Submitted filename: Review PLOS One-2.docx [file pone.0230290.s001.docx]

Editor Comments

This is a retrospective study in which the authors evaluate the effect of propofol vs desflurane anesthesia on survival. Propofol anesthesia improved overall survival in robot-assisted radical prostatectomy compared with desflurane anesthesia. Furthermore, patients with propofol anesthesia had less postoperative biochemical recurrence.

This is an interesting study adding to literature that supports the beneficial effects of propofol on overall survival and cancer recurrence.

I am concerned about the statistics. First of all, the authors did not distinguish between mediators and confounders in their analysis, which means that thy might have over-adjusted.

Second, I do not see any correction for multiple comparisons.

The manuscript is difficult to read because the authors use many abbreviations but also because the English has to be corrected.

Author Comments

This is a retrospective study in which the authors evaluate the effect of propofol vs desflurane anesthesia on survival. Propofol anesthesia improved overall survival in robot-assisted radical

prostatectomy compared with desflurane anesthesia. Furthermore, patients with propofol anesthesia had less postoperative biochemical recurrence.

This is an interesting study adding to literature that supports the beneficial effects of propofol on overall survival and cancer recurrence. However, I am concerned about the statistics. First of all the authors did not distinguish between mediators and confounders in their analysis, which means that thy might have over-adjusted. Second, I do not see any correction for multiple comparisons.

Specific comments

Abstract:

Methods: please specify the outcomes

Page 8: the authors use many abbreviations, which makes the manuscript hard to read

Introduction

Page 9, para 1: please change to: Unfortunately, recurrence of prostate cancer after surgery increases postoperative morbidity and mortality.

Page 9, para 1: which may impair cell-mediated…………

Page 10 para 1: Please move the discussion in regards to neuraxial anesthesia to the discussion section. It inhibits the flow of the introduction.

Page 10, para 1, hypothesis: please be more specific. What is your primary and what is the secondary hypothesis?

Page 11, para 2: “The exclusion criteria were propofol anesthesia combined with VAs or regional analgesia” I assume that regional anesthesia was excluded for the desflurane patients as well?

Page 11, last para: did you really use 100% oxygen?

Page 12, para 2: “time since earliest included patient”. What does that mean? Why did you not use “year of surgery”?

Page 13, para 1: you list many potential confounding factors. Please be careful in distinguishing whether these variables are true confounders or mediators. A confounder is associated with both the treatment as well as the outcome, while a mediator is associated with the outcome.

F.e Tumor staging is associated with the outcome (survival) but not with the treatment (propofol or desflurane anesthesia) and thus is a mediator for which you cannot adjust.

Page 13, para 2: The primary outcome was overall survival? Or was it time to death?

Page 13: the authors make several comparisons. How did you correct for multiple comparisons?

Page 14, para 3: “according to the anesthetic technique and other variables were compared individually in a” What are the other variables?

Table 1: Please use standardized difference for the matched variables (and not p-value)

Page 18, limitations: as you excluded patients with regional anesthesia your fith limitation is actually not a limitation.

Please be consistent with the tense (past or present) you use throughout the manuscript.
