# Supplementary material for: Propofol-based total intravenous anesthesia is associated with better survival than desflurane anesthesia in robot-assisted radical prostatectomy
Source: PLoS One. 2020 Mar 17;15(3):e0230290. doi: 10.1371/journal.pone.0230290 (PMC7077845; doi:10.1371/journal.pone.0230290)
Supplement: Attachment — Submitted filename: Reply_template_PONE_R1.doc [file pone.0230290.s002.doc]

**Reply to the reviewers’ comments**

| Reviewer Number | Original comments of the reviewer | Reply by the author(s) | Changes done on page number and line number |
| --- | --- | --- | --- |
| 1 (Overall) | 1. The authors were diligent with comparison between subtypes, including TNM classification and histology. Unfortunately, this meant small groups for propensity matching which influences the rigorousness of their statistical significance. | We appreciate your comments. PS matching may minimize possible confounding in this observational study (J Am Heart Assoc 2018;7:e008010). However, the small groups for propensity matching may influence the rigorousness of the statistical significance in our study. Fortunately, regardless the analytic approaches were applied, the point estimates and significances of relative risk of propofol versus desflurane were similar. | P20, Line 15-19. |
| 1 (Overall) | 2. There was wide variability in the HR for overall survivability, but results were still meaningful. | We appreciate your comments. The wide confidence intervals mainly due to low mortality rate. |  |
| 1 (Overall) | 3. Their overall conclusion that results were significant but require further investigation is appropriate. | We appreciate your comments, and further prospective multi-center study is warranted. | P20, Line 15-19. |
| 1 (Copyedits) | 1. Abstract conclusion: Remove "Besides", substitute "In addition,". | We have revised the manuscript as you suggested. | P7, Line 2. |
| 1 (Copyedits) | 2. Introduction, page 9, line 6. Remove "Besides," | We have revised the manuscript as you suggested. | P9, Line 7. |
| 1 (Copyedits) | 3. Introduction, page 9, Line 9-10, change to: Unfortunately, recurrence of prostate cancer in patients after surgery results in... | We have revised the manuscript as you and the Reviewer 2 suggested. | P9, Line 9-10. |
| 1 (Copyedits) | 4. Introduction, page 9, line 10-11: change to "Surgical intervention itself may result in neuroendocrine and metabolic changes which may lead to impaired cell-mediated immunity and activation of circulating tumor cell implantation." | We have revised the manuscript as you suggested. | P9, Line 11-12. |
| 1 (Copyedits) | 5. Introduction, page 9, line 15: Change to "Research has shown that volatile anesthetics are pro-inflammatory..." | We have revised the manuscript as you suggested. | P9, Line 18-19. |
| 1 (Copyedits) | 6. Introduction, page 6, line 6: change to "We hypothesized that patients under desflurane anesthesia may have poorer outcomes than patients under propofol..." | We have revised the manuscript as you and the Reviewer 2 suggested. | P10, Line 2-4. |
| 1 (Copyedits) | 7. In Methods section, page 11, same units should be used for flow: 300 mL/min | We have revised the manuscript as you suggested. | P12, Line 1. |
| 1 (Copyedits) | 8. Page 13, please change to: "Because significant interactions with the two anesthetic techniques (propofol or desflurane) were found, we also performed subgroup analyses for TNM stage as well as postoperative BCR." Please add detail to statement: Because significant interactions with the two anesthetic techniques (propofol or desflurane) were found. | Thanks for your remind. We found it was a wrong statement. According to Table 3, there were no interactions with the two anesthetic techniques (propofol or desflurane) for TNM stage (p=0.926).  We have revised the manuscript. | 1. P13, Line 18-20. 2. Table 3. |
| 1 (Copyedits) | 9. page 14, end of page: "This finding did not change substantially in the multivariable analyses after adjustment for those significant variables in the univariable analyses and 9 surgeons (HR, 0.12; 95% CI, 0.03–0.54; P = 0.006)". Do you mean after adjustment for significant variables including the nine surgeons? | Adjusted-HRs were adjusted by those variables were significant in the univariable analyses and surgeons (n = 9). Functional status was excluded from the multivariable due to it was highly correlated with ASA. Postoperative biochemical recurrence was another outcome, therefore, it was also not included. | Table 2(footnotes). |
| 1 (Copyedits) | 10. page 15, "After the multivariable analysis, a higher preoperative PSA level was another variable that was identified that significantly increased the mortality risk. | We have revised the manuscript as you suggested. | P16, Line 3-5. |
| 1 (Copyedits) | 11. page 15, end of page: In summary, patients with desflurane anesthesia had more significant disease progression (such as postoperative BCR) than those with propofol anesthesia. | We have revised the manuscript as you suggested. | P17, Line 1-2. |
| 1 (Copyedits) | 12. Page 16, in discussion: "The major findings in the present study are that propofol anesthesia in RARP improves survival and reduces postoperative BCR compared with desflurane anesthesia. The results were consistent with our previous reports demonstrating that propofol anesthesia..." | We have revised the manuscript as you suggested. | P18, Line 2-4. |
| 1 (Copyedits) | 13. Page 16, discussion: "The result was consistent with Coughlin et al., who reported that..." | We have revised the manuscript as you suggested. | P18, Line 13-14. |
| 1 (Copyedits) | 14. Page 16, discussion: "However, previous data indicated that considerable number of men had  prostate cancer despite being within normal PSA range. Importantly, many of these patients were later found to have high grade histology." | We have revised the manuscript as you suggested. | P18, Line 17-19. |
| 1 (Copyedits) | 15. Page 16, discussion, end of page: "The perioperative stress induced by surgery leads to metabolic and neuroendocrine changes that result in significant depression of cell mediated immunity. This can result in the emergence of micro-seed tumor cells during surgery, which can avoid..." | We have revised the manuscript as you suggested. | P18, Line 21-23. |
| 1 (Copyedits) | 16. Page 17, discussion:" In addition, HIF-1 was overexpressed in pancreatic cancer,32 and a knockout of HIF-1..." | We have revised the manuscript as you suggested. | P19, Line 17. |
| 1 (Copyedits) | 17. Page 18, discussion: " However, the cancer-specific mortality was lower than previous reports (1.7% and 4.3%). This might be due to the higher surgical volume at our center and higher level of experience because we excluded patients from the learning curve period of the first two years of surgery performed by our teams." | We have revised the manuscript as you suggested. | P20, Line 8-11. |
| 1 (Copyedits) | 18. Page 19, discussion: "We conducted the surgeon analysis (n=9) and the result showed that, in our high volume surgery center, the postoperative outcome was not affected by which surgeon performed the procedure (Table 2). Finally, the anesthesiologists chose the type of anesthesia, which may have been subject to original selection bias..." | We have revised the manuscript as you suggested. | P21, Line 8-11. |
| 2 (Overall) | 1. This is an interesting study adding to literature that supports the beneficial effects of propofol on overall survival and cancer recurrence. However, I am concerned about the statistics. First of all, the authors did not distinguish between mediators and confounders in their analysis, which means that thy might have over-adjusted. Second, I do not see any correction for multiple comparisons. | Thank you for your valuable suggestions.  1. We have changed confounders to covariates since confounders have more restricted definition.  2. Regardless how many covariates have been adjusted, the findings did not alter substantially. It means that over-adjustment maybe not in issue.  3. There were no serious multiple comparison problems in the statistical methods used. | P13, Line 7. |
| 2 (Specific) | 1. Abstract:  Methods: please specify the outcomes | The primary outcome was overall survival, and the secondary outcome was postoperative biochemical recurrence. | P6, Line 12-13. |
| 2 (Specific) | 2. Page 8: the authors use many abbreviations, which makes the manuscript hard to read | We have deleted 7 abbreviations in the revised manuscript. | P8. |
| 2 (Specific) | 3. Page 9, para 1: please change to: Unfortunately, recurrence of prostate cancer after surgery increases postoperative morbidity and mortality. | We have revised the manuscript as you suggested. | P9, Line 9-10. |
| 2 (Specific) | 4. Page 9, para 1: which may impair cell-mediated………… | We have revised the manuscript as you suggested. | P9, Line 11-12. |
| 2 (Specific) | 5. Page 10 para 1: Please move the discussion in regards to neuraxial anesthesia to the discussion section. It inhibits the flow of the introduction. | We have revised the manuscript as you suggested. | P19, Line 6-10. |
| 2 (Specific) | 6. Page 10, para 1, hypothesis: please be more specific. What is your primary and what is the secondary hypothesis? | 1. Primary hypothesis: patients under desflurane anesthesia may have poorer overall survival than patients under propofol anesthesia.  2. Secondary hypothesis: patients under desflurane anesthesia may have more postoperative biochemical recurrences than patients under propofol anesthesia. | P10, Line 2-4. |
| 2 (Specific) | 7. Page 11, para 2: “The exclusion criteria were propofol anesthesia combined with VAs or regional analgesia” I assume that regional anesthesia was excluded for the desflurane patients as well? | Yes, regional anesthesia/analgesia was excluded for the desflurane patients as well. | P11, Line 13-14. |
| 2 (Specific) | 8. Page 11, last para: did you really use 100% oxygen? | We did use FiO2 of 100% oxygen in the two groups intraoperatively. (Anesthesiology 2018;129:932-941, Br J Anaesth. 2019;123:151-160, PLoS One 2019;14:e0224728.) |  |
| 2 (Specific) | 9. Page 12, para 2: “time since earliest included patient”. What does that mean? Why did you not use “year of surgery”? | Time since the earliest included patient (a continuous variable), which served as a surrogate of the calendar year (categorical variable). We prefer to use a continuous variable which provides easier information (mean and SD) for readers rather than a categorical variable. Our multivariable model did not include time since the earliest included patient because it was not significant in the univariable analysis. When the calendar period was further adjusted in the multivariable model, the HR (95% CI) became 0.084 (0.02-0.40). This did not change our conclusion. |  |
| 2 (Specific) | 10. Page 13, para 1: you list many potential confounding factors. Please be careful in distinguishing whether these variables are true confounders or mediators. A confounder is associated with both the treatment as well as the outcome, while a mediator is associated with the outcome. | Please refer to our response to Reviewer 2 (overall). |  |
| 2 (Specific) | 11. F.e Tumor staging is associated with the outcome (survival) but not with the treatment (propofol or desflurane anesthesia) and thus is a mediator for which you cannot adjust. | Please refer to our response to Reviewer 2 (overall). |  |
| 2 (Specific) | 12. Page 13, para 2: The primary outcome was overall survival? Or was it time to death? | The primary outcome was overall survival. |  |
| 2 (Specific) | 13. Page 13: the authors make several comparisons. How did you correct for multiple comparisons? | Please refer to our response to Reviewer 2 (overall). |  |
| 2 (Specific) | 14. Page 14, para 3: “according to the anesthetic technique and other variables were compared individually in a” What are the other variables? | Other variables are time since the earliest op, age, PSA level, Charlson comorbidity index, functional status, ASA, TNM stage, tumor grade, tumor size, intraoperative blood transfusion, grade of surgical complications, postoperative radiation therapy, postoperative androgen deprivation therapy, and Postoperative biochemical recurrence (univariable model in Table 2). In addition, Adjusted-HRs were adjusted by those variables were significant in the univariable analyses and surgeons (n = 9). Functional status was excluded from the multivariable due to it was highly correlated with ASA. Postoperative biochemical recurrence was another outcome, therefore it was also not included (multivariable model in Table 2). | Table 2. |
| 2 (Specific) | 15. Table 1: Please use standardized difference for the matched variables (and not p-value) | We have added standardized mean differences (SMD) in Table 1. | Table 1. |
| 2 (Specific) | 16. Page 18, limitations: as you excluded patients with regional anesthesia your fith limitation is actually not a limitation. | Thank for your kindly reminder, we have deleted regional anesthesia as the limitation. |  |
| 2 (Specific) | 17. Please be consistent with the tense (past or present) you use throughout the manuscript. | We have revised the manuscript as you suggested. |  |
